# Passive Heat Exposure Alters Perception and Executive Function

**DOI:** 10.3389/fphys.2018.00585

**Published:** 2018-05-25

**Authors:** Rachel A. Malcolm, Simon Cooper, Jonathan P. Folland, Christopher J. Tyler, Caroline Sunderland

**Affiliations:** ^1^Department of Sport Sciences, Sport, Health and Performance Enhancement Research Centre, Nottingham Trent University, Nottingham, United Kingdom; ^2^School of Sport, Exercise and Health Sciences, Loughborough University, Loughborough, United Kingdom; ^3^Department of Life Sciences, University of Roehampton, London, United Kingdom

**Keywords:** cognitive function, passive, hyperthermia, perceptual responses, core temperature

## Abstract

Findings regarding the influence of passive heat exposure on cognitive function remain equivocal due to a number of methodological issues including variation in the domains of cognition examined. In a randomized crossover design, forty-one male participants completed a battery of cognitive function tests [Visual Search, Stroop, Corsi Blocks and Rapid Visual Information Processing (RVIP) tests] prior to and following 1 h of passive rest in either hot (39.6 ± 0.4°C, 50.8 ± 2.3% Rh) or moderate (21.2 ± 1.8°C, 41.9 ± 11.4% Rh) conditions. Subjective feelings of heat exposure, arousal and feeling were assessed alongside physiological measures including core temperature, skin temperature and heart rate, at baseline and throughout the protocol. Response times were slower in the hot trial on the simple (main effect of trial, *P* < 0.001) and complex (main effect of trial, *P* < 0.001) levels of the Stroop test (Hot: 872 ± 198 ms; Moderate: 834 ± 177 ms) and the simple level of the visual search test (Hot: 354 ± 54 ms; Moderate: 331 ± 47 ms) (main effect of trial, *P* < 0.001). Participants demonstrated superior accuracy on the simple level of the Visual Search test in the hot trial (Hot: 98.5 ± 3.1%; Moderate: 97.4 ± 3.6%) (main effect of trial, *P* = 0.035). Participants also demonstrated an improvement in accuracy on the complex level of the visual search test following 1 h passive heat exposure (Pre: 96.8 ± 5.9%; Post: 98.1 ± 3.1%), whilst a decrement was seen across the trial in the moderate condition (Pre: 97.7 ± 3.5; Post: 97.0 ± 5.1%) (time^*^trial interaction, *P* = 0.029). No differences in performance were observed on the RVIP or Corsi Blocks tests (all *P* > 0.05). Subjective feelings of thermal sensation and felt arousal were higher, feeling was lower in the hot trial, whilst skin temperature, core temperature and heart rate were higher (main effects of trial, all *P* < 0.001). The findings of the present study suggest that response times for perception and executive function tasks are worse in the heat. An improvement in accuracy on perceptual tasks may suggest a compensatory speed-accuracy trade-off effect occurring within this domain, further highlighting the task dependant nature of heat exposure on cognition.

## Introduction

While the negative effects of heat exposure on physical performance are well known (Drust et al., 2005; Morris et al., 2005), data regarding the effects of heat exposure on cognitive performance are less well understood. It is important to understand the effects of heat exposure across a range of domains of cognition, given that the components of cognition will interact to affect overall performance in sport, however also in professions such as firefighting and the military (Allard and Burnett, 1985; Hemmatjo et al., 2017). Gaoua et al. (2018) has recently provided valuable insight into the mechanisms associated with changes in cognition in response to passive heat exposure, however an understanding across a broad range of cognitive domains is still lacking.

The impact of heat exposure on cognitive function remains equivocal, largely due to a number of methodological discrepancies across the research, such as the cognitive domain tested. Heat exposure has been reported to have a positive effect on attention (Simmons et al., 2008; Lee et al., 2014; Watkins et al., 2014; Schlader et al., 2015) and working memory (Bandelow et al., 2010; Lee et al., 2014), but a negative effect on working memory capacity (Racinais et al., 2008; Gaoua et al., 2011; Liu et al., 2013) and no effect on perception (Gaoua et al., 2011), short term memory (Wijayanto et al., 2013) or aspects of attention (Sun et al., 2013). The type and timing of cognitive test utilized and the mode, intensity and duration of heat exposure (Gaoua, 2010) directly influence the perceptual strain and physiological strain experienced by an individual. Additionally, the use of exercise-induced heating protocols prevents the isolation of heat as a stressor (Racinais et al., 2008). These variables likely contribute to the discrepancies in the literature. This provides rational for a protocol where as many confounding variables (e.g., low intensity exercise) are eradicated and a breadth of cognitive domains can be tested under the same conditions using a large cohort of the same participants.

Traditionally, an elevation in core temperature has been highlighted as a mediator of the heat induced negative effects on cognitive function, detrimentally influencing working memory (Lieberman et al., 2005; Morley et al., 2012), executive function (Schlader et al., 2015) and attention (Lieberman et al., 2005; Simmons et al., 2008; Schlader et al., 2015). However, the negative subjective feelings and large increases in skin temperature, without associated increases in core temperature (Gaoua et al., 2012), in response to passive heating, can also negatively influence cognition (Ramsey and Kwon, 1992; Racinais et al., 2008). When thermal strain is increased performance on complex tasks have generally demonstrated greater vulnerability to the detrimental effects of heat (Hancock, 1989), whilst simple task performance can be maintained. The influence of factors such as skin temperature and subjective feeling changes on cognitive function in response to heat exposure, prior to increases in core temperature, warrant further investigation.

Consequently, the aim of this study was to examine the impact of passive heat exposure on cognitive function, eradicating the confounding influence of exercise, whilst examining the influence of rapid changes in skin temperature. It is hypothesized that passive heat exposure will adversely influence complex cognitive tasks, particularly those in the domains of executive function and attention, an effect mediated by negative subjective feelings.

## Methods

Forty one healthy active males were recruited to take part. The mean (± SD) age, height and body mass of the 41 participants who completed the study were 21.3 ± 1.6 years, 181.0 ± 5.7 cm and 81.6 ± 9.8 kg, respectively. This study was approved by Nottingham Trent ethical advisory committee. In line with the declaration of Helsinki, all participants volunteered to participate following a detailed explanation of the study, and provided written informed consent and a health screen prior to participation to ensure participants were in good health.

### Study design

All data were collected at Nottingham Trent University, located in the UK, between the months of September and December (2015 and 2016), e.g., winter months, to prevent any heat acclimatization effect (Nakamura and Okamura, 2001). Each participant completed a familiarization trial, a control trial in a thermoneutral environment (21.2 ± 1.8°C and 41.9 ± 11.4% Rh) and a hot trial in the environmental chamber (39.6 ± 0.4°C and 50.8 ± 2.3% Rh) in a randomized crossover design. Each experimental trial was separated by exactly 7 d and performed at the same time of day to eradicate any influence of circadian rhythm (Van Dongen and Dinges, 2005). Participants avoided strenuous exercise 24 h before each main trial and completed a food diary in the 24 h prior to the first main trial, which was replicated prior to the second main trial. On the day of each main trial participants were asked to arrive at the laboratory at 9 a.m. and 2 h postprandial, having abstained from caffeine and following the consumption of 500 ml of water ~2 h prior to arrival at the laboratory.

### Protocol

#### Familiarization

The familiarization trial was completed 7 d prior to the first main experimental trial. The protocol was explained to participants, who were given the opportunity to ask questions and familiarize themselves with the equipment being used. The participant's height (Seca 123, Seca Ltd.) and body mass (GFK 150 AEADAM digital scale, Vitech scientific Ltd.) was measured. Participants completed a full battery of the cognitive function tests (detailed below) to negate any learning effects (Cooper et al., 2015b).

### Main trial

**Figure 1:**
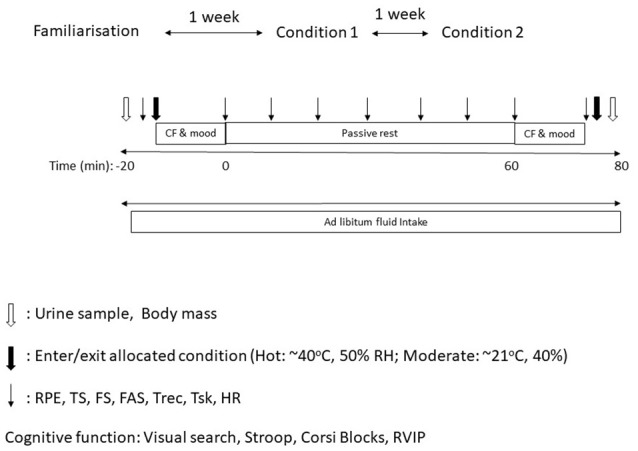
Protocol diagram. 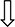 Urine sample and body mass. 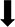 Enter/ exit allocated condition. ↓ RPE, TS, FAS, FS, Trec, Tsk, & HR. 
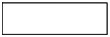
 CF & mood, cognitive function and mood questionnaire; RPE, rating of perceived exertion; TS, thermal sensation; FS, feeling scale; FAS, felt arousal scale; Tc, core temperature; Tsk, skin temperature.

On arrival, participants recorded their nude body mass and self-inserted a rectal probe. In both trials the first physiological measures were taken in the laboratory (21.2 ± 1.8°C and 41.9 ± 11.4% Rh) before participants entered their allocated condition. This set of measurements will be referred to as baseline measures. Participants then entered their allocated condition and completed the first battery of cognitive function tests and a mood questionnaire, followed by 1 h seated rest in their allocated condition, prior to completing the second battery of cognitive function tests and mood questionnaire, also in the allocated condition. Participants were instructed to remain seated throughout the 1 h rest and not engage with any electronic devices. Throughout all trials participants were allowed to drink water ad libitum (~4°C). All water drank and urine produced was weighed in order to establish body mass lost and estimated sweat rate. All perceptual and thermal measures were taken at baseline, every 10 min for the duration of the passive rest and on completion of each battery of cognitive tests (Figure 1).

### Measurements

#### Mood questionnaire

Participants completed the Brunel Mood Scale (BRUMS) questionnaire (Terry et al., 1999) answering 24 items related to 6 aspects of mood; anger, confusion, depression, fatigue, tension and vigor. A scale of 1 to 5 was used to assess each of these items (where 1: “not at all,” 2 “a little,” 3: “moderately,” 4: “quite a lot,” 5: “extremely”), which enabled a score out of 20 to be calculated for each aspect of mood.

#### Cognitive function tests

The cognitive test battery lasted approximately 15 min and was administered via a laptop computer (Thinkpad T450, Lenovo PC HK Limited, China) at −20 min (immediately on entering the allocated condition) and following 1 h passive rest in the allocated condition. The four cognitive tests used were Visual search test, Stroop test, Corsi Blocks and rapid visual information processing (RVIP) and were completed in that order on each occasion. All cognitive tests for each trial were completed in the allocated condition. Participants wore noise-canceling headphones in order to eradicate any distracting stimuli. Prior to each test, 3–6 practice stimuli were presented during which feedback was provided on the accuracy of the response. The purpose of these practice stimuli was to eradicate any learning effect, and the results for practice stimuli were not included in analysis. The reliability of a number of these tests (Visual search and the Stroop test) has previously been assessed (CV = 6.68% and CV = 4.32%, respectively; Cooper et al., 2015b).

#### Perception (visual search test)

Perception and visual processing were assessed using the Visual Search test, as used by Cooper et al. (2015a). The test consisted of two levels, each containing 21 stimuli. On the simple level of this test participants were required to respond to the appearance of a bold, solidly outlined green triangle. The complex level of the test was also made up of 21 stimuli, however required participants to respond to the appearance of a triangle shape made up of a number of dots. The background was comprised of green dots covering the screen, which were redrawn every 250 ms to induce the visual effect of a flickering background. For both levels of the test the stimuli appeared at randomized locations across the screen at variable intervals. Participants were instructed to press the space bar in response to the stimulus as quickly as possible on both test levels. This test examines how well participants can filter distracting information from their surroundings and interpret specific cues, utilizing visual processing and perception. The response time between the presentation of a stimulus and the response was recorded as well as the proportion of the correct responses achieved.

#### Executive function (stroop test)

The Stroop test is an executive function and selective attention task which measures frontal lobe function (Stroop, 1935) and the ability to suppress an automated response. The test is comprised of two levels, which have varying levels of interference. Each level involves a test word appearing in the center of the screen, with a target word and a distractor either side of it. The target word's position was counterbalanced for the left and right side within each test level and the participant was asked to respond as quickly as possible, using the left and right arrow key, to identify the target word's position.

The simple level of the test had 20 stimuli and the complex level was made up of 40. On the simple level of the Stroop test, the target word was the word matching the word in the center of the screen, with all words presented in white font. The color interference complex level of the test required the participant to select the word which corresponds with the color the word in the center of the screen was written in, rather than the word itself (which was an incongruent color). The inter-stimulus interval was 1 s, and choices remained on the screen until a response was selected. Response time between the presentation of the stimuli and the response was recorded and the proportion of correct responses was measured.

#### Working memory (corsi blocks)

The Corsi Blocks test assessed visio-spatial short term working memory (Corsi and Michael, 1972). A 3 × 3 grid filled the screen, where the squares randomly lit up. Participants were required to then replicate the order of the squares lighting up by clicking on the boxes. The sequence length started at three and with each correct response, the sequence got longer in length by one unit. Where participants correctly recalled a sequence of 9 boxes, the grid increased in size to 4 × 4. Performance was determined by the mean of the 3 longest correctly remembered sequences (Cooper et al., 2016).

#### Attention (rapid visual information processing (RVIP)

The RVIP test assesses sustained attention as used by Hogervorst et al. (2008). The test lasted 5 min and numbers 2–9 appear on the screen for the duration at 600 ms intervals, with 8 target sequences appearing per min. Participants were instructed to press the space bar each time a sequence of 3 odd or even numbers were shown e.g., “2-4-6,” “3-5-7,” and “4-6-8.” Responses could be registered during the last digit sequence and the 1,500 ms that followed. Response time from the presentation of the stimulus to the response was recorded, alongside the proportion of correct responses.

#### Physiological measures

All physiological measurements were taken at baseline, every 10 min during seated rest and at the completion of the each cognitive test. Heart rate monitor belts (Polar, T31 Coded Transmitter, Kempele, Finland) were worn for the duration of each trial and heart rate was recorded from a Polar watch (Polar, FT1 Polar, Finland). Core temperature was measured via rectal probe (MEAS 440 Series Temperature Probe, Measurement Specialities Inc., USA) self-inserted 10 cm beyond the anal sphincter. Core temperature was measured throughout the procedure using the core temperature logger (4600 Thermometer, Measurement Specialities Probe, Ohio, USA). Skin temperature was measured at the midpoint on the right thigh, using a hand held infrared gun (Standard ST-812 InfraRed Thermometer, CEM, Shenzhen, China). Urine samples were analyzed for urine osmolality using a handheld osmometer (Pocket-Pal Osmo-Osmocheck^TM^, 4595-E04, Vitech-Scientfic Ltd, Horsham, UK) at the beginning and end of each trial. Any value <800 mosmol.kg^−1^ was considered hydrated (Perrier et al., 2015).

#### Subjective feelings

Rating of perceived exertion (RPE) (Borg, 1962) was measured on a 6-20-point scale to measure perception of effort. Feeling was measured using a −5 (Very bad) to +5 (Very good) scale (Hardy and Rejeski, 1989). Arousal was measured using a 1 (low-arousal) to 6 (high-arousal) scale (Svebak and Murgatroyd, 1985). Thermal sensation (TS) was measured using a 0 (Unbearably cold) to 8 (Unbearably hot) scale (Young et al., 1987).

#### Data analysis

Physiological data, perceptual measures and Corsi blocks data were analyzed using SPSS (Version 23, SPSS Inc., Chicago, Il, USA) via two-way repeated measures Analysis of Variance (ANOVA), using a trial (2 factor: moderate and hot) by session time (2 for Corsi and 7 time points for physiological and perceptual data) approach. Where paired comparisons were required, paired samples *t*-tests with Bonferroni corrections were conducted for physiological measures only.

The cognitive data (Stroop, visual search and RVIP) were analyzed using R (www.r-project.org). Linear mixed effects models were used to analyse the data, with a random effect for each participant. Response time analyses were performed using the nlme package, which implements mixed effects models and produces T statistics. Accuracy analyses were performed with the lme4 package, to account for the binomial outcome data distribution, which produces z statistics. All analyses were conducted using a trial by session time interactions. Separate analyses for each test level on the Stroop test and the visual search test were completed to account for the varying cognitive demands of these tests (Miyake et al., 2000). Response times on all cognitive tests were log transformed to normalize the distribution, which demonstrated the right hand skew expected of human response times. Minimum and maximum cut-offs were employed in order to exclude any responses which could be deemed anticipatory or delayed. Therefore, across the Visual Search and Stroop tests response times less than 100 ms were excluded and maximum cut off times were 1,500 and 3,000 ms, for the simple and complex levels, respectively. The minimum cut off time for RVIP was 200 ms and the maximum was 1,500 ms. Only the response times of correct responses were used for response time analysis.

The effect size (Cohen's *d*) of all significant differences were calculated using trial pairings and interpreted using the following thresholds: <0.2 = trivial effect; 0.2 – <0.5 = small effect; 0.5–0.8 = moderate effect and >0.8 = large effect (Cohen, 1992). For all analyses, significance was set as *P* < 0.05. Data are presented as mean ± standard deviation.

## Results

### Cognitive function

Mean data for all cognitive tests are presented in Table 1.

**Table 1:** Cognitive function data [mean ± SD; range (min, max)] across the hot and moderate trials.

**Test**	**Variable**	**Test level**	**Moderate**		**Hot**		**Trial effect**	**Time effect**	**Interaction**	**Effect size**
			**Pre**	**Post**	**Pre**	**Post**				
Visual Search	Response Time (ms)	Simple	330 ± 46 213 (222, 457)	333 ± 47 216 (243, 439)	349 ± 46 191 (274, 465)	359 ± 61 265 (262, 527)	*P* < 0.01*	*P* < 0.01*	*P* = 0.85	*d* = 0.46 small
		Complex	1,213 ± 268 1,245 (902, 2,147)	1,180 ± 240 1,010 (838, 1,848)	1,270 ± 281* 1,164 (791, 1,955)	1,307 ± 300* 1,257 (890, 2,147)	*P* = 0.17	*P* = 0.28	*P* = 0.01*	*d* = 0.33 small
	Accuracy (%)	Simple	97.9 ± 3.1 12.5 (87.5, 100)	96.9 ± 4.0 16 (84, 100)	98.9 ± 3.7 22.2 (77.8, 100)	98.1 ± 2.3 4.5 (95.5, 100)	*P* = 0.04*	*P* = 0.53	*P* = 0.53	*d* = 0.33 small
		Complex	97.7 ± 3.5 12.5 (87.5, 100)	97.0 ± 5.1 22.2 (77.8, 100)	96.8 ± 5.9 27.6 (72.4, 100)	98.1 ± 3.1 12.5 (87.5, 100)	*P* = 0.14	*P* = 0.25	*P* = 0.03*	*d* = 0.0 trivial
Stroop test	Response Time (ms)	Simple	618 ± 74 294 (488, 782)	638 ± 95 418 (501, 919)	665 ± 105* 455 (460, 915)	657 ± 130* 630 (500, 1,130)	*P* < 0.01*	*P* < 0.01*	*P* < 0.01*	*d* = 0.33 small
		Complex	842 ± 182 764 (567, 1, 331)	826 ± 174 683 (596, 1,279)	894 ± 196 803 (614, 1,417)	851 ± 200 1,011 (565, 1,576)	*P* < 0.01*	*P* = 0.15	*P* = 0.14	*d* = 0.20 small
	Accuracy (%)	Simple	98.9 ± 3.0 14.3 (85.7, 100)	97.0 ± 4.5 14.3 (85.7, 100)	97.9 ± 4.3 14.3 (85.7, 100)	97.3 ± 4.4 14.3 (85.7, 100)	*P* = 0.23	*P* = 0.03*	*P* = 0.22	*d* = 0.10 trivial
		Complex	94.8 ± 6.1 25 (75, 100)	95.1 ± 4.8 15 (85, 100)	96.3 ± 4.2 15 (85, 100)	94.0 ± 5.8 25 (75, 100)	*P* = 0.14	*P* = 0.66	*P* = 0.22	*d* = 0.04 trivial
Corsi Blocks	Sequence length		6.0 ± 0.7 2.8 (4.4, 7.2)	6.1 ± 0.9 4.2 (4, 8.2)	5.9 ± 0.8 3.8 (3.6, 7.4)	5.9 ± 0.9 3.8 (4.2, 8)	*P* = 0.22	*P* = 0.71	*P* = 0.74	*d* = 0.13 trivial
RVIP	Response Time (ms)		496 ± 82 419 (263, 682)	486 ± 61 264 (383, 647)	494 ± 77 433 (339, 772)	506 ± 57 236 (404, 640)	*P* = 0.99	*P* = 0.20	*P* = 0.57	*d* = 0.13 trivial
	Accuracy (%)		52.8 ± 18.3 75 (15, 90)	56.9 ± 19.8 76 (17, 93)	53.0 ± 16.2 64 (26, 90)	55.7 ± 16.9 68 (15, 83)	*P* = 0.46	*P* < 0.01*	*P* = 0.27	*d* = 0.06 trivial

Cohen's d effect size for the hot vs. moderate condition.

* Indicates significant at P < 0.05 level.

* *Indicates significantly different from the moderate trial at P < 0.05 level*.

### Perception (visual search)

#### Response times

##### Simple

Overall, response times were slower in the hot trial [main effect of trial, *t*_(1, 3687)_ = 4.9, *P* < 0.01; *d* = 0.46, small effect], and response times slowed over time [main effect of time, *t*_(1, 3687)_ = 2.8, *P* < 0.01]. However the pattern of change in response times across the hot and moderate trial was similar (trial^*^time interaction, *P* = 0.85).

##### Complex

Response times were not different between the hot and moderate trial (main effect of trial, *P* = 0.17; *d* = 0.33, small effect) and there was no change across time (main effect of time, *P* = 0.28). However the pattern of change across trials was different, whereby responses slowed in the heat, whereas they improved in the moderate condition [trial^*^time interaction, *t*_(3, 3687)_ = 2.5, *P* = 0.01].

#### Accuracy

##### Simple

Participants demonstrated superior accuracy on the simple level of the test in the hot trial [main effect of trial, z_(1, 3844)_ = 2.1, *P* = 0.04; *d* = 0.33, small effect]. However, there was no effect of time (main effect of time, *P* = 0.53) on accuracy and the pattern of change in accuracy across the hot and moderate trials did not differ (trial^*^time interaction, *P* = 0.53).

##### Complex

Accuracy did not differ between trials, (main effect of trial, *P* = 0.14; *d* = 0.0, trivial effect) or across time (main effect of time, *P* = 0.25). However, accuracy improved on the complex level of the test following 1 h of passive heat exposure, whereas a decrement occurred in the moderate condition [trial^*^time interaction, *z*_(3, 3872)_ = 2.2, *P* = 0.03].

### Executive function (stroop test)

#### Response times

##### Simple

Overall, response times were slower in the hot trial [main effect of trial, *t*_(1, 2376)_ = 5.8, *P* < 0.01; *d* = 0.33, small effect] and changed across time [main effect of time, *t*_(1, 2376)_ = 2.9, *P* < 0.01]. The pattern of change differed between trials, where a marginal improvement in response time was seen following passive heating, while the control trial saw a slowing in response time following 60 min exposure to moderate conditions [trial^*^time interaction, *t*_(1, 2376)_ = −2.6, *P* < 0.01].

##### Complex

Overall, response times were slower in the hot trial [main effect of trial, *t*_(1, 3297)_ = 4.7, *P* < 0.01; *d* = 0.20, small effect], however response time did not change across time (main effect of time, *P* = 0.15) and the pattern of change between trials did not differ (trial^*^time interaction, *P* = 0.14).

#### Accuracy

For both the simple and complex levels of the Stroop test accuracy did not differ between the hot and moderate trials (simple - main effect of trial, *P* = 0.23, *d* = 0.10, trivial effect; complex—main effect of trial, *P* = 0.14, *d* = 0.13, trivial effect). Accuracy decreased across time (main effect of time, *P* = 0.03) on the simple level of the test in both trials, however was unaffected in the complex level (main effect of time, *P* = 0.66). For both levels. the pattern of change across the hot and moderate trials was similar (simple - trial^*^time interaction, *P* = 0.22; complex – trial^*^time interaction, *P* = 0.22).

##### Working memory (corsi blocks)

The mean of the 3 longest remembered sequences did not differ between trials (main effect of trial, *P* = 0.22, *d* = 0.13, trivial effect), or across time (main effect of time, *P* = 0.71). The pattern of change across the hot and moderate trials was similar (trial^*^time interaction, *P* = 0.74).

### Attention (RVIP)

#### Response times

Response times were not different between the hot and moderate trials (main effect of trial, *P* = 0.99, *d* = 0.13, trivial effect), and did not differ across time (main effect of time, *P* = 0.20). The pattern of change across the hot and moderate trials was similar (trial^*^time interaction, *P* = 0.57).

#### Accuracy

Overall, there was no effect of trial on accuracy (*P* = 0.46, *d* = 0.06, trivial effect), and the pattern of change across trials was not different (*P* = 0.27). However, across time accuracy improves [main effect of time, z_(3, 8790)_ = 2.7, *P* < 0.01].

### Physiological data

#### Rectal temperature

**Figure 2:**
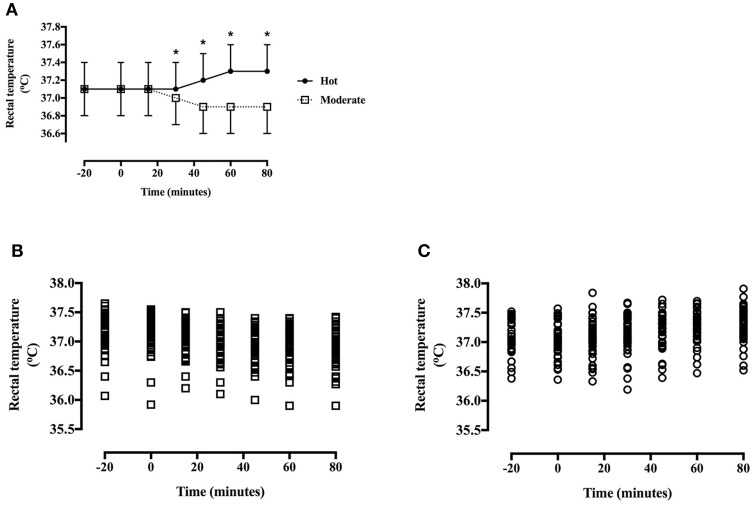
**(A)** Rectal temperature during the hot and moderate trials (mean ± SD). Main effect of trial: *P* < 0.001; main effect of time: *P* = 0.61; trial*time interaction: *P* < 0.001. *Identifying time point where rectal temperature is significantly greater in the hot trial (*P* < 0.05). **(B)** Individual data points for core temperature in the moderate trial. **(C)** Individual data points for core temperature in the hot trial.

Rectal temperature was greater in the hot trial [main effect of trial, *F*_(1, 39)_ = 10.2, *P* < 0.01, *d* = 0.49, small effect; Figure 2], however no main effect of time was seen (*P* = 0.61). A trial^*^time interaction was seen for core temperature [*F*_(4, 137)_ = 37.7, *P* < 0.001]. Rectal temperature was not different at baseline, 0 and 15 min, however rectal temperature was greater in the heat at every time point thereafter (all *P* < 0.05; Figure 2).

#### Skin temperature

**Figure 3:**
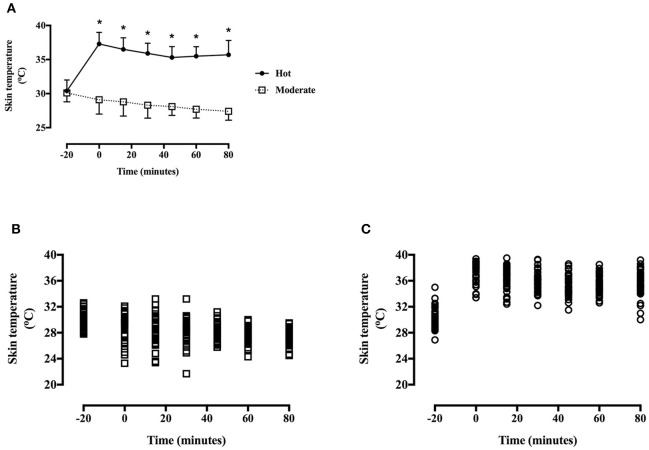
**(A)** Skin temperature during the hot and moderate trials (mean ± SD). Main effect of trial: *P* < 0.001; main effect of time: *P* < 0.001; trial*time interaction: *P* < 0.001. *Identifying time points where skin temperature is significantly greater in the hot trial (*P* < 0.05). **(B)** Individual data points for skin temperature in the moderate trial. **(C)** Individual data points for skin temperature in the hot trial.

Thigh skin temperature was greater in the hot trial [main effect of trial, *F*_(1, 40)_ = 1008.9, *P* < 0.001, *d* = 2.99, large effect] (Figure 3), increased across time [main effect of time, *F*_(4, 172)_ = 35.4, *P* < 0.001] and the pattern of change differed between trials [trial^*^time interaction, *F*_(4, 172)_ = 93.2, *P* < 0.001; Figure 3]. Skin temperature did not differ at baseline [*T*_(40)_ = 1.5, *P* = 0.15], however was greater in the heat at all other time points (all *P* < 0.01).

#### Heart rate

**Figure 4:**
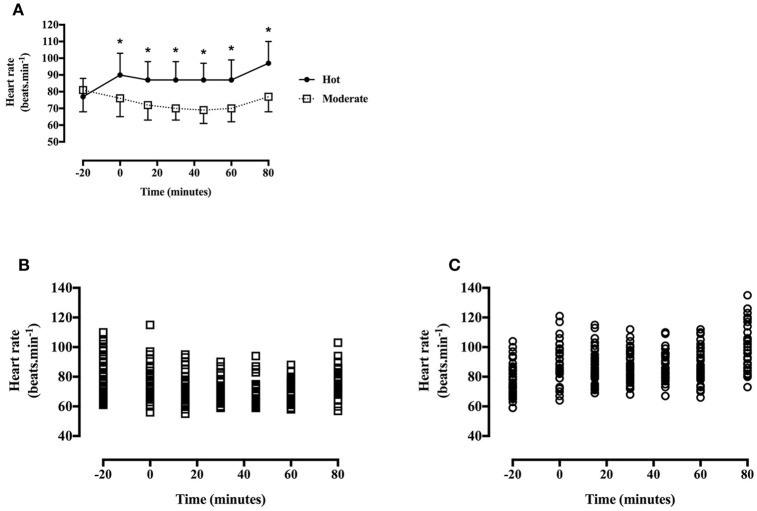
**(A)** Heart rate during the hot and moderate trials (mean ± SD). Main effect of trial: *P* < 0.001; main effect of time: *P* < 0.001; Tt, trial*time interaction: *P* < 0.001. *Identifying time point where heart rate is significantly greater in the hot trial (*P* < 0.05). **(B)** Individual data points for heart rate in the moderate trial. **(C)** Individual data points for heart rate in the hot trial.

Heart rate was greater during the hot trial [main effect of trial, *F*_(1, 40)_ = 139.0, *P* < 0.001, *d* = 1.21, large effect; Figure 4], and increased throughout the trial in the hot trial whereas a gradual decrease was seen in the moderate trial [time^*^trial interaction, *F*_(6, 240)_ = 27.4, *P* < 0.001]. Heart rate did not differ at baseline [*t*_(40)_ = −1.9, *P* = 0.07], however was greater at every subsequent time point in the hot trial (all *P* < 0.01).

#### Hydration status

Participants were hydrated (<800 mosmol.kg^−1^) at the beginning (Hot: 683 ± 297 mosmol.kg^−1^; Control 630 ± 310 mosmol.kg^−1^) and end (Hot: 394 ± 273 mosmol.kg^−1^, Control: 387 ± 219 mosmol.kg^−1^) of each trial. There was no effect of trial (*P* = 0.43) or a trial^*^time interaction (*P* = 0.38) for urine osmolality, however participants became more hydrated across time [main effect of time, *F*_(1, 40)_ = 65.381, *P* < 0.01] in both trials. *Ad libitum* water intake was 444 ± 278 ml in the moderate trial and 1,060 ± 553 ml in the hot trial.

There was no effect of trial (*P* = 0.70), time (*P* = 0.91) or an interaction effect (*P* = 0.42) on body mass. Sweat rate was greater in the heat (hot: 0.56 ± 0.38 l.h^−1^, control: 0.25 ± 0.20 l.h^−1^, *P* < 0.01), however body mass change, corrected for fluid intake and urine output, was maintained in both trials (hot: −0.94 ± 0.83%, control: – 0.31 ± 0.52%).

#### Subjective feelings

Rating of perceived exertion [main effect of trial, *F*_(1, 40)_ = 29.4, *P* < 0.01, *d* = 0.92, large effect], felt arousal [main effect of trial, *F*_(1, 39)_ = 9.9, *P* < 0.01, *d* = 0.42, small effect] and thermal sensation [main effect of trial, *F*_(1, 40)_ = 156.0, *P* < 0.01, *d* = 2.41, large effect] were higher in the heat, whereas feeling [main effect of trial, *F*_(1, 40)_ = 16.7, *P* < 0.01, *d* = 0.58, moderate effect] was lower in the hot trial (Table 2).

**Table 2:** Perceived ratings of exertion, thermal sensation, feeling and felt arousal during the hot and moderate trials [mean ± SD; range (min, max)].

**Trial**	**Time (min)**							**Effect size**
	**−20**	**0**	**15**	**30**	**45**	**60**	**80**	
**Rating of perceived exertion (RPE) (T, Tt)**								
Moderate	6.0 ± 0.2 1 (6, 7)	6.2 ± 0.6 3 (6, 9)	6.1 ± 0.3 1 (6, 7)	6.2 ± 0.5 3 (6, 9)	6.1 ± 0.5 3 (6, 9)	6.2 ± 0.7 3 (6, 9)	6.3 ± 0.8 3 (6, 9)	*d* = 0.92 large
Hot	6.1 ± 0.3 2 (6, 8)	7.5 ± 1.7* 6 (6, 12)	7.6 ± 2.0* 7 (6, 13)	7.9 ± 2.3* 7 (6, 13)	8.0 ± 2.4* 8 (6, 14)	8.1 ± 2.6* 8 (6, 14)	8.7 ± 2.8* 9 (6, 15)	
**Thermal sensation (TS) (T, Tt)**								
Moderate	3.3 ± 0.8 3 (1, 4)	3.4 ± 0.8 4 (1, 5)	3.4 ± 0.8 3 (2, 5)	3.4 ± 0.8 3.5 (1.5, 5)	3.3 ± 0.8 2.5 (2, 4.5)	3.2 ± 0.9 3 (2, 5)	3.1 ± 0.9 4 (1.5, 5.5)	*d* = 2.41 large
Hot	3.5 ± 0.9 4.5 (2, 6.5)	5.6 ± 0.6* 3 (4, 7)	5.9 ± 0.6* 3 (4, 7)	6.1 ± 0.7* 3 (4, 7)	6.2 ± 0.7* 3.5 (4, 7.5)	6.2 ± 0.8* 3.5 (4, 7.5)	6.4 ± 0.8* 3.5 (4, 7.5)	
**Feeling scale (FS) (T)**								
Moderate	1.8 ± 1.6 6 (−1, 5)	1.5 ± 1.5 6 (−1, 5)	1.7 ± 1.5 5 (0, 5)	1.8 ± 1.4 5 (0, 5)	1.8 ± 1.4 5 (0, 5)	1.7 ± 1.5 6 (−1, 5)	1.6 ± 1.4 5 (0, 5)	*d* = 0.58 moderate
Hot	1.2 ± 1.7 8 (−3, 5)	0.7 ± 1.6 9 (−4, 5)	0.9 ± 1.7 6 (−2, 4)	0.7 ± 2.0 7 (−3, 4)	0.7 ± 1.9 7 (−3, 4)	0.6 ± 1.9 7 (−3, 4)	0.5 ± 1.7 7 (−3, 4)	
**Felt arousal scale (FAS) (T)**								
Moderate	1.8 ± 1.1 4 (1, 5)	1.9 ± 1.1 3 (1, 4)	1.9 ± 1.1 3 (1, 4)	1.8 ± 1.1 3 (1, 4)	1.8 ± 1.1 3 (1, 4)	1.7 ± 1.1 3 (1, 4)	1.9 ± 1.1 3 (1, 4)	*d* = 0.42 small
Hot	2.0 ± 1.1 4 (1, 5)	2.5 ± 1.1 3 (1, 4)	2.3 ± 1.1 4 (1, 5)	2.3 ± 1.1 4 (1, 5)	2.1 ± 1.4 7 (−3, 4)	2.2 ± 1.1 3 (1, 4)	2.5 ± 1.2 4 (1, 5)	

T, main effect of trial: P < 0.05, t, main effect of time: P < 0.05; Tt, trial*time interaction: P < 0.05.

* *Indicates significantly different from the moderate trial at P < 0.05 level*.

A trial^*^time interaction was seen for rating of perceived exertion [*F*_(2, 90)_ = 17.2, *P* < 0.01] and thermal sensation [*F*_(2, 98)_ = 44.5, *P* < 0.01], however the pattern of change didn't differ between trials for FS (*P* = 0.10) and FAS (*P* = 0.12). Thermal sensation and rating of perceived exertion did not differ at baseline, however were both greater in the heat at the remainder of time points (all *P* < 0.01).

#### Mood questionnaire

Anger, tension and vigor were all unaffected by the heat. Confusion [main effect of trial, *F*_(1, 31)_ = 4.6, *P* = 0.04], depression [main effect of trial, *F*_(1, 31)_ = 6.6, *P* = 0.02] and fatigue [main effect of trial, *F*_(1, 31)_ = 4.2, *P* < 0.05], were greater in the hot trial compared to the moderate trial. A significant time^*^trial interaction was seen for fatigue [*F*_(1, 31)_ = 21.433, *P* < 0.01], whereby fatigue increased on the hot trial [*t*_(31)_ = −4.6, *P* < 0.01].

## Discussion

Passive heat exposure has a detrimental influence on response times in perception tasks; however a trade-off appeared to occur whereby accuracy improves. Response times on the simple and complex levels of the executive function task were slower at both time points in the heat, suggesting an impairment of more complex cognitive functions. Whereas, no change was observed in performance for the working memory or attention task.

### Perception

There is limited research investigating the effects of passive heating on visual perception, and particularly visual search. The findings of the present study therefore provide novel findings on this specific test. Results demonstrated slower response times at both time points in the heat for the simple and complex (small effect) perception task. In addition, for the complex visual perception test a slowing of response times, alongside an improvement in accuracy was evident in the hot environment. This is in contrast to the findings of Gaoua et al. (2011) where a match to sample visual search test was unaffected by heat exposure, but in line with the study of Hancock and Dirkin (1982), where accuracy improved at the expense of a slowing in response time when cortical temperature was increased. The slower response times in the heat for the complex level of the visual search test agrees with the research suggesting that more complex tasks may be affected by hyperthermia, whilst simpler tasks will be unaffected (Gaoua et al., 2011). These findings are in line with previous literature where decrements in complex cognitive tasks have been observed following 5 min of heat exposure, where skin temperature increased by ~3°C but core temperature was unaffected (Gaoua et al., 2012). fMRI data from Liu et al. (2013) suggests hyperthermia increases activation in the temporal lobe during a visual perception task, whereas a decrease in activity was seen in neurons in the frontal lobe, parietal lobe and occipital lobe. These findings suggest the brain alters the distribution of resources in response to passive heat exposure in an attempt to maintain performance in this domain, helping explain the speed-accuracy trade-off in the present study.

In the present study, participants reported lower levels of pleasure (feeling scale data) and greater confusion, depression and fatigue (BRUMS questionnaire data) in the hot trial. This coincided with a significant increase in skin temperature and thermal strain, potentially explaining these negative subjective feelings. The compensatory responses, such as an increase in heart rate as shown in the present study, and an increase in sweat rate, that occur in response to heat to prevent large changes in core temperature may also influence subjective feelings and contribute to decrements in cognitive function, rather than core temperature change itself (Gaoua et al., 2012).

### Working memory

In the present study, no effect of passive heat exposure was seen on visuo-spatial memory. Wijayanto et al. (2013) found that passive heat exposure, via lower leg immersion, had no effect on short-term memory. Interestingly, this study showed an increase in oxygen delivery to the prefrontal lobe in the heat, suggesting an increase in the recruitment of neural resources in order to maintain performance. Gaoua et al. (2011) and Racinais et al. (2008) found a significant decrement on working memory when employing a more intense heating strategy (15 min walking followed by 45 min rest in 50°C and 50% Rh), which resulted in a greater increase in core temperature. Physical exertion is known to influence cognitive function (Hogervorst et al., 1996; McMorris and Graydon, 1997). Although the time course of cognitive recovery is unknown post exercise, it is possible this difference in mode of heating may have influenced Gaoua et al. (2011) and Racinais et al. (2008) results, hence the findings were not truly a result of “passive” heat stress. Therefore, despite inducing a greater increase in core temperature, the stress exerted on participants is not truly from a “passive” protocol (Simmons et al., 2008; Gaoua et al., 2011). The present study addresses this concern by using a passive heating protocol.

### Executive function

Response times on the simple and complex level of the executive function (Stroop) task were slower at both time points in the heat compared to the moderate trial. This agrees with Liu et al. (2013), who found that executive function was the primary domain to be influenced in the heat. The executive attention network allows an individual to decipher between potentially incongruent responses, in order to select the correct response. Liu et al. (2013), utilizing fMRI imaging, found activation of the prefrontal cortex differed in the heat, an area related to the efficiency of resolving conflict between stimuli and responses. Hence this may explain the overall slower response times on both levels of the Stroop task. This finding contradicts those of Gaoua et al. (2018), who found changes in only complex tasks, explained by alterations in EEG activity. Gaoua et al. (2018) suggest that during simple tasks neural resources can compensate for the added strain to prevent a detriment to performance. However, the current study implies that when heat exposure is truly passive, both simple and complex tasks are at risk, an effect likely mediated by negative subjective feelings. This is in agreement with previous findings showing the negative influence of subjective state on cognitive processes (Gaoua et al., 2012).

### Attention

The current study suggests that short duration heat exposure does not influence sustained attention. Neave et al. (2004) found moderately intense exercise-induced hyperthermia negatively impacted response times for attention, whereas Gaoua et al. (2011) found that very low intensity exercise stress and passive heating had no effect on RVIP performance, other than an increase in false alarms. Neave et al. (2004) achieved a lower core temperature than Gaoua et al. (2011), therefore the variation in exercise stress is more likely the cause of changes in cognitive function. Wohlwend et al. (2017) highlights the differing responses in cognition across exercise intensities, hence highlighting the need to control for exercise if heat is the variable under consideration. The findings of Schlader et al. (2015) also agree with those of the present study where heat exposure, incurred through a water-perfused suit, had no effect on sustained visual attention. Similar to this study no meaningful change in core temperature was observed, however subjective feelings were detrimentally affected. Therefore, collectively these findings suggest that changes in attention may require a greater disruption to the body's state of homeostasis, suggesting resources can be redistributed to protect function within this domain. Liu et al. (2013) found that brain areas associated with the alerting network of attention were activated more during passive hyperthermia (premotor cortex, middle temporal gryus and superior parietal lobule) yet no change in performance was seen. This may suggest that in the present study increased activation within these areas has enabled the maintenance of attention during passive hyperthermia.

### Limitations

The findings of the present study can only be applied to the cognitive domains tested. Further, an additional time point for cognitive function could have been employed, allowing core temperature to increase further. This may have allowed a greater understanding of how both skin and core temperature changes affect cognitive function over a more prolonged period. However, we were specifically interested in short-term effects and mental fatigue may have affected the reliability of the data. The use of only one site for the measurement of skin temperature does not give a whole body measure, however enabled the most accurate measurement in a short period of time. Finally, using a standardized order for the cognitive function tests may have caused mental fatigue, further exacerbated by greater discomfort in the later tests.

## Conclusion

The main finding of the present study suggests that neural resources may be sacrificed in certain domains of cognition (e.g., perception and executive function) in order to maintain performance in others (e.g., memory and attention). The detrimental effects seen on perception and executive function may be explained by an alliesthesial effect caused by changes in subjective feelings and skin temperature. The present study provides novel findings regarding the effects of passive heat exposure (without the confounding effects of dehydration and exercise) across a range of domains of cognitive function.

## Author contributions

RM, CS, SC, CT, and JF assisted with study design and manuscript preparation and editing. RM and CS completed data collection and RM and SC completed data analysis. All authors read and approved the final manuscript.

### Conflict of interest statement

The authors declare that the research was conducted in the absence of any commercial or financial relationships that could be construed as a potential conflict of interest.
